# Enhancing Payment Integrity in U.S. Healthcare Through Value-Based Care: The Role of Bundled Episodes and Alternative Payment Models

**DOI:** 10.7759/cureus.101825

**Published:** 2026-01-19

**Authors:** Amol Kodan

**Affiliations:** 1 Public Health, Monroe University, New York City, USA

**Keywords:** alternative payment model, episode based care, healthcare reimbursement, payment integrity, utilization management, value based care

## Abstract

Improper payments, unnecessary utilization, and administrative inefficiencies represent persistent structural challenges within the United States healthcare system. This places significant pressure on healthcare spending. Value-based care (VBC), particularly through bundled episode payments and alternative payment models (APMs), has emerged not only as a potential cost containment strategy but also as a potential framework for strengthening payment integrity and promoting appropriate resource use.

This article examines how value-based care models, particularly bundled payments and APMs, strengthen payment integrity by addressing structural drivers of improper payments through improved transparency, accountability, and stewardship of healthcare resources across the care continuum. Bundled episode payments establish a single, prospectively defined payment for all services associated with a discrete episode of care, supporting greater predictability of spending and improved coordination across settings, while reducing fragmentation within the care continuum.

By shifting reimbursement away from volume-based incentives toward outcome-oriented accountability, VBC facilitates clearer evaluation of utilization patterns and enables earlier identification of unexplained variation through routine monitoring and analytics. These design features complement traditional oversight and utilization management efforts by addressing the underlying payment and delivery-system drivers of fee-for-service (FFS) reimbursement.

This technical report synthesizes existing evidence and policy analysis to examine how value-based care models address a critical policy gap by embedding payment integrity and utilization oversight within payment design, rather than relying solely on retrospective enforcement.

## Introduction

Payment integrity challenges in healthcare are closely associated with the incentive structure of the prevailing fee-for-service (FFS) payment model, which reimburses providers based on the volume and intensity of services delivered rather than overall value or outcomes [1]. This structure can contribute to service fragmentation, increased coding intensity, duplicative testing, and the delivery of low-value care [1,2]. Patients frequently receive care without advance clarity regarding total episode costs, while services are billed separately across providers and settings. This fragmentation obscures the actual cost of care, limits meaningful patient engagement, and reduces accountability for utilization patterns. In such environments, distinguishing inappropriate billing or utilization from legitimate clinical variation can be challenging when relying solely on traditional oversight mechanisms [1].

In response, advancing value-based care as a foundational payment reform could serve as a model for federal and other health policies [3]. Under value-based care models, payment is linked to predefined quality measures and total cost accountability, altering the economic context in which care is delivered. Bundled episode payments and APMs are central to this model and have been implemented across both public and private sectors, including through initiatives led by the Centers for Medicare & Medicaid Services [2,3]. Rather than relying solely on retrospective detection tools, these models seek to address structural drivers of inappropriate utilization by aligning incentives prospectively [4].

Payment integrity refers to ensuring that healthcare payments are accurate, appropriate, and consistent with applicable coverage, coding, and billing rules. Improper payments include overpayments, underpayments, or payments made in error, regardless of intent, while fraud and abuse involve intentional or prohibited actions designed to obtain unwarranted reimbursement. Unnecessary utilization refers to services that provide limited or no clinical value but may still meet technical billing requirements. Distinguishing these concepts is important, as payment integrity challenges may arise from structural incentives and system design rather than fraudulent behavior alone.

Efforts to improve transparency through price disclosure and consumer-facing tools play an important role but have limited impact when implemented without accompanying payment reform [3]. When reimbursement remains volume-based, price visibility alone may not meaningfully influence utilization decisions. In contrast, value-based payment frameworks define costs prospectively and assign clear financial accountability, supporting more effective stewardship of healthcare resources [4].

These design features have essential implications for payment integrity. When episode costs are fixed and accountability is shared, opportunities for billing inflation are constrained, and variation in utilization becomes more readily observable [2,5]. Comparative analysis across providers enables the identification of abnormal spending patterns and unexplained variation more efficiently. In this way, value-based care complements existing program integrity and oversight efforts by enhancing transparency, predictability, and accountability across care delivery [1,5].

Methods

This manuscript is a technical report based on a narrative synthesis of peer-reviewed literature, federal policy documents, and published evaluations of bundled payment and APM initiatives. For the purpose of policy analysis, sources were included based on relevance to payment design, utilization oversight, and payment integrity mechanisms.

## Technical report

Structural drivers of improper payments in fee-for-service systems

Payment integrity challenges in the United States healthcare system are closely linked to the structural features of the FFS model, which fragments care delivery and billing across providers, settings, and services [1,2]. Under FFS, each service is billed independently, often by multiple clinicians involved in the same episode of care. This approach generates complex and disaggregated claims data that can obscure the total scope, cost, and appropriateness of care delivered for a given clinical condition. As a result, inappropriate, duplicative, or unsupported services may be embedded within otherwise legitimate claims, making them difficult to distinguish from expected clinical variation through routine claims review alone [1,3].

Oversight challenges are particularly pronounced in service categories involving multiple care transitions or documentation-intensive billing [3]. Some examples for this, deriving from clinical care, can include post-acute care, imaging, durable medical equipment, and procedural services, which can possibly result in a disproportionate share of improper payments. These domains require coordination across providers and rely heavily on accurate coding and documentation, thereby increasing the risk of improper payments when oversight mechanisms are limited or delayed. FFS reimbursement can amplify these risks by directly tying payment to service volume rather than outcomes or episode-level value, reducing short-term financial disincentives for excessive or low-value utilization [1,4].

Because FFS generates large volumes of individual claims, program integrity efforts often rely on retrospective audits, sampling, and post-payment review. While necessary, these approaches are resource-intensive and may identify issues only after expenditures have occurred. Consequently, payment integrity efforts in FFS environments are frequently reactive, focusing on recovery and correction rather than prevention [1,3].

Bundled payments, risk adjustment, and observability in value-based models

Bundled episode payments and other value-based payment models address several of the structural limitations of FFS by defining payment prospectively for a complete episode of care [5]. By consolidating related services into a single payment, bundled models reduce fragmentation and establish clearer expectations regarding the scope, duration, and cost of care for specific clinical conditions. This prospective structure enhances transparency and supports more meaningful evaluation of utilization patterns across providers and settings [2,6,7].

When reimbursement is fixed at the episode level, opportunities for billing inflation are reduced, as providers do not receive additional payment for increasing the number of individual services delivered within an episode. Instead, financial performance depends on effective care coordination, avoidance of unnecessary services, and management of total episode costs. Importantly, these models incorporate risk adjustment methodologies, such as those used in Medicare payment systems, to account for differences in patient complexity and clinical risk, thereby supporting fair comparisons across providers.

The episode-level payment model also improves observability for payers and oversight entities. Rather than assessing isolated services, analysts can evaluate total episode costs, utilization intensity, and outcomes against expected benchmarks, facilitating earlier identification of unexplained variation [2,7]. These signals can inform targeted monitoring and review while reducing reliance on broad, service-level audits. In this way, bundled payment models complement existing program integrity and oversight activities by improving transparency, predictability, and accountability across care delivery, without replacing traditional audit or enforcement mechanisms [1,5].

Episode-level benchmarking, analytics, and payment integrity

A defining feature of VBC is the use of episode-level benchmarking and analytics to evaluate performance across providers delivering comparable care. By comparing costs, utilization patterns, and outcomes for standardized episodes, payers can establish expected performance ranges at regional and national levels [2,7]. Providers whose episode costs or service intensity consistently exceed these benchmarks may be identified as outliers for further review.

Episode-level benchmarking supports payment integrity by narrowing the range of expected billing behavior and reducing ambiguity around acceptable utilization. Because providers are aware that their performance will be assessed relative to peers managing similar patient populations, incentives favor adherence to evidence-informed care pathways and avoidance of unnecessary services [4,5]. Advanced analytics further enhance this process by enabling near real-time monitoring of episode performance, allowing the identification of emerging patterns of overutilization or cost escalation earlier than under traditional retrospective claims review [7,8].

When variation in cost or utilization cannot be explained by patient complexity, risk adjustment, or clinical need, concerns regarding inappropriate billing or abusive utilization may warrant closer examination [6]. By aggregating services into coherent episodes rather than dispersing them across disconnected claims, VBC improves the visibility of such deviations and supports more targeted, timely responses through existing oversight mechanisms (Table 1).

**Table 1:** Comparative impact of fee-for-service and value-based care models on payment integrity and utilization oversight

Dimension	Fee for Service Model	Value-Based Care Bundles and APMs
Payment Structure	Separate payment for each individual service	Single prospective payment for an entire episode
Care Coordination	Fragmented across providers and settings	Encourages coordination across the care continuum
Cost Predictability	Low predictability with variable cumulative costs	High predictability through predefined benchmarks
Transparency of Utilization	Limited visibility across fragmented claims	Fully transparent with prospectively defined, upfront episode level costs
Risk of Improper Payments	Higher due to coding intensity, duplication, and fragmentation	Reduced through consolidated payment and shared accountability
Oversight Approach	Largely, retrospective audits and post payment review	Prospective design with continuous performance monitoring
Observability of Variation	Difficult to distinguish clinical variation from inappropriate use	Easier identification of unexplained variation via benchmarking
Role of Analytics	Claims level analysis after payment	Near real time episode and system level analytics
Provider Accountability	Limited to individual billed services	Shared financial and quality accountability
Spillover Effects	Minimal impact beyond billed services	Standardization of care pathways across broader patient populations
Fraud and Abuse Exposure	Higher exposure to embedded inappropriate services	Structural constraints reduce billing inflation opportunities
Policy Implications	Reactive recovery focused integrity efforts	Proactive prevention-oriented integrity framework

## Discussion

Alternative payment models and shared risk accountability

APMs extend transparency and accountability beyond individual episodes by holding participating entities responsible for the total cost and quality of care across defined populations [4]. Under these models, spending targets and quality benchmarks are established prospectively, creating clear expectations for performance and supporting more predictable care pathways for members. Providers and payers assume shared responsibility for managing utilization risk rather than relying on fragmented, service-level billing characteristic of FFS reimbursement [5,9].

Shared risk arrangements alter financial incentives that otherwise enable inappropriate utilization. When revenue is no longer directly linked to service volume, excessive or low-value care does not generate additional payment and may instead reduce performance margins. As a result, organizations participating in APMs rely less on post-payment claim denial processes and more on prospective contract design, internal utilization management, and continuous performance monitoring [4,9]. This alignment reduces administrative complexity and supports more proactive approaches to payment integrity.

System-level analytics and early identification of aberrant utilization

APMs enable system-level analytics that strengthen oversight and early identification of aberrant utilization patterns. Population-based cost and utilization data allow comparisons across providers, regions, and time periods, making deviations from expected norms more readily observable [7,8]. Providers or delivery systems whose performance consistently diverges from benchmarks may be flagged for further evaluation before improper payments accumulate.

This analytic transparency shifts payment integrity efforts from predominantly retrospective enforcement toward proactive risk management. Rather than relying solely on audits after payments are made under the FFS, payers can monitor performance continuously and address emerging risks in near real time. Clearly defined benchmarks and performance thresholds reduce uncertainty in distinguishing inappropriate utilization from legitimate clinical variation [6,8]. These capabilities enhance, rather than replace, existing audit and enforcement functions.

Spillover effects and market-wide transparency

Adoption of VBC models can produce spillover effects extending beyond formally contracted populations. Providers participating in bundled payments and APMs often standardize care pathways, documentation practices, and utilization controls across their broader patient base, reflecting organizational investments in analytics and governance that persist irrespective of payer or payment model [5,7].

As VBC penetration increases nationally, market norms shift toward greater transparency and accountability. Highly aggressive FFS billing practices become less sustainable as providers align operational strategies with value-based incentives. These diffusion effects may reinforce reductions in inappropriate utilization and improve care efficiency even in settings where FFS reimbursement remains in use, contributing to broader system-level improvements [4,9].

Limitations and policy implications

VBC models enhance transparency and reduce structural vulnerabilities to improper payments; they do not eliminate all forms of fraud or abuse. Eligibility fraud, identity misuse, and collusive behaviors occurring outside defined care episodes remain challenges. In addition, complex risk adjustment methodologies require ongoing oversight to prevent gaming or unintended distortion of performance comparisons [6,10,11]. Nonetheless, these risks are generally more tractable than those inherent in fragmented FFS systems.

From a policy perspective, bundled episode payments and APMs should be viewed as core components of a national strategy to strengthen payment integrity, enhance transparency, and promote responsible stewardship of healthcare resources. By defining costs prospectively, assigning shared financial accountability to providers and payers, and enabling clearer analytic identification of abnormal utilization patterns, VBC addresses structural conditions that allow inappropriate billing to persist. Continued, carefully designed expansion of these models offers a pathway toward a more transparent, efficient, and financially sustainable healthcare system.

In addition, the prospective design of APMs supports clearer role delineation among payers, providers, and oversight entities, reducing reliance on retrospective adjudication alone. By embedding expectations for cost, quality, and utilization management into contract structures, these models encourage earlier internal correction of emerging issues before they rise to the level of formal enforcement. While continued monitoring and refinement are necessary, particularly with respect to risk adjustment and performance measurement, this approach underscores how payment reform can function as a supportive infrastructure for payment integrity rather than a substitute for statutory oversight. Collectively, these features reinforce the role of APMs as a pragmatic, system-oriented mechanism for improving transparency and accountability in U.S. healthcare financing.

Future empirical evaluations demonstrating reductions in audited error rates, decreases in unexplained episode-level variation, or improved anomaly detection yield would be necessary to confirm these effects. Until such evidence is available, this analysis provides a conceptual framework for understanding how payment design may support more proactive and efficient stewardship of healthcare resources.

## Conclusions

Transitioning from FFS reimbursement to VBC represents a structural shift that has the potential to transform how healthcare spending is conducted, better organized, monitored, and governed. By defining costs prospectively, aggregating services into episodes, and aligning payment with quality and total cost accountability, VBC and APMs may reduce fragmentation and improve transparency across care delivery. Through these mechanisms, VBC is expected to strengthen payment integrity by making abnormal utilization patterns more visible and easier to evaluate within existing oversight frameworks. While no payment model eliminates all forms of fraud or abuse, the expansion of VBC may serve as a policy approach that plausibly supports more proactive, data-informed stewardship of healthcare resources rather than a demonstrated outcome in the absence of primary empirical analysis. It could possibly shift the system towards a scalable pathway that is more efficient, accountable, and sustainable in the U.S. healthcare system. Future evaluations demonstrating reductions in audited error rates or decreases in unexplained episode-level cost variation would provide stronger empirical support for these expectations and are needed to clarify the impact of VBC on payment integrity.

## References

[1] Berwick DM, Hackbarth AD (2012). Eliminating waste in US health care. JAMA.

[2] Leusder M, Porte P, Ahaus K, van Elten H (2022). Cost measurement in value-based healthcare: a systematic review. BMJ Open.

[3] (2026). Centers for Medicare & Medicaid Services: Fiscal year 2024 improper payments fact sheet. https://www.cms.gov/newsroom/fact-sheets/fiscal-year-2024-improper-payments-fact-sheet.

[4] Song Z, Rose S, Safran DG (2014). Changes in health care spending and quality 4 years into global payment. N Engl J Med.

[5] Mechanic RE, Altman SH (2009). Payment reform options: episode payment is a good place to start. Health Aff (Millwood).

[6] Pope GC, Kautter J, Ellis RP (2004). Risk adjustment of Medicare capitation payments using the CMS-HCC model. Health Care Financ Rev.

[7] Hussey PS, Mulcahy AW, Schnyer C, Schneider EC (2012). Closing the quality gap: revisiting the state of the science (vol. 1: bundled payment: effects on health care spending and quality). Evid Rep Technol Assess (Full Rep).

[8] Liao JM, Navathe AS, Werner RM (2020). The impact of Medicare's alternative payment models on the value of care. Annu Rev Public Health.

[9] Howard SW, Bradford N, Belue R (2024). Building alternative payment models in health care. Front Health Serv.

[10] Joynt Maddox KE, Orav EJ, Zheng J, Epstein AM (2018). Evaluation of Medicare's bundled payments initiative for medical conditions. N Engl J Med.

[11] Rajkumar R, Conway PH, Tavenner M (2014). CMS--engaging multiple payers in payment reform. JAMA.

